# Dual Activity BLEG-1 from *Bacillus lehensis* G1 Revealed Structural Resemblance to B3 Metallo-β-Lactamase and Glyoxalase II: An Insight into Its Enzyme Promiscuity and Evolutionary Divergence

**DOI:** 10.3390/ijms22179377

**Published:** 2021-08-29

**Authors:** Shaw Xian Au, Nur Syazana Dzulkifly, Noor Dina Muhd Noor, Hiroyoshi Matsumura, Raja Noor Zaliha Raja Abdul Rahman, Yahaya M. Normi

**Affiliations:** 1 Enzyme and Microbial Technology (EMTech) Research Center, Faculty of Biotechnology and Biomolecular Sciences, Universiti Putra Malaysia, Serdang 43400, Selangor, Malaysia; aushawxian@gmail.com (S.X.A.); dina@upm.edu.my (N.D.M.N.); rnzaliha@upm.edu.my (R.N.Z.R.A.R.); 2 Department of Cell and Molecular Biology, Faculty of Biotechnology and Biomolecular Sciences, Universiti Putra Malaysia, Serdang 43400, Selangor, Malaysia; syazanadzulkifly@gmail.com; 3 Department of Biochemistry, Faculty of Biotechnology and Biomolecular Sciences, Universiti Putra Malaysia, Serdang 43400, Selangor, Malaysia; 4 College of Life Sciences, Ritsumeikan University, Noji-Higashi, Kusatsu 525-8577, Japan; h-matsu@fc.ritsumei.ac.jp; 5 Department of Microbiology, Faculty of Biotechnology and Biomolecular Sciences, Universiti Putra Malaysia, Serdang 43400, Selangor, Malaysia

**Keywords:** BLEG-1, metallo-β-lactamase, glyoxalase II, enzyme promiscuity, evolutionary divergence, crystal structure, active-site loop

## Abstract

Metallo-β-lactamases (MBLs) are class B β-lactamases from the metallo-hydrolase-like MBL-fold superfamily which act on a broad range of β-lactam antibiotics. A previous study on BLEG-1 (formerly called Bleg1_2437), a hypothetical protein from *Bacillus lehensis* G1, revealed sequence similarity and activity to B3 subclass MBLs, despite its evolutionary divergence from these enzymes. Its relatedness to glyoxalase II (GLXII) raises the possibility of its enzymatic promiscuity and unique structural features compared to other MBLs and GLXIIs. This present study highlights that BLEG-1 possessed both MBL and GLXII activities with similar catalytic efficiencies. Its crystal structure revealed highly similar active site configuration to YcbL and GloB GLXIIs from *Salmonella enterica*, and L1 B3 MBL from *Stenotrophomonas maltophilia*. However, different from GLXIIs, BLEG-1 has an insertion of an active-site loop, forming a binding cavity similar to B3 MBL at the N-terminal region. We propose that BLEG-1 could possibly have evolved from GLXII and adopted MBL activity through this insertion.

## 1. Introduction

Metallo-hydrolase-like MBL-fold superfamily of proteins is a large and ancient group of proteins that are widely distributed over the kingdoms of bacteria, archaea, and eukarya. Sequence, structure, and biochemical studies revealed that the superfamily consists of members with diverse functions, consisting of class B β-lactamase, glyoxalase II (GLXII), N-acyl-L-homoserine lactonase (AHL), persulfide dioxygenase, flavodiiron protein, choline-binding protein, cleavage and polyadenylation specificity factors, arylsulfatase, 5′-exonuclease, ribonuclease, cyclic nucleotide phosphodiesterase, insecticide hydrolase, and proteins required for natural transformation competence [1,2,3,4]. Members of this superfamily contain conserved αβ/βα metallo-β-lactamase-fold (MBL-fold) domain, with two central β-sheets surrounded by solvent-exposed α-helices, and either have a mononuclear or binuclear metal binding site located at the interface of the β/β sandwich, characterized by the His-Xaa-His-Xaa-Asp-His motif [5]. Most of them bind to zinc ion(s), although the ability to bind to several metal ions such as Zn^2+^, Fe^2+^, Mn^2+^, and Mg^2+^ were reported as well [1,6]. Interestingly, enzyme promiscuity among some members being able to catalyze side reactions resembling the activity of other family members has been reported. This include the human metallo-hydrolase-like MBL-fold proteins MBLAC2 involved in B-cell exosome biogenesis; and SNM1A and SNM1B exonucleases acting as DNA cross-link repair proteins, all of which exhibited nitrocefin and penicillin hydrolysis activity [7] apart from their central roles mentioned above. In addition to this is the novel PNGM-1 B3 MBL identified from deep sea metagenome exhibiting both MBL and tRNase Z activities [8]. Apart from the reported native dual activities of metallo-hydrolase superfamily members, directed evolution studies have demonstrated that an enzyme can be converted to resemble another family member with different enzymatic activity. A case in point is the mutational study performed on human glyoxalase II (GLXII), which resulted in the introduction of MBL activity to the enzyme with total abolishment of its native GLXII activity [9]. This highlights that mechanistically diverse enzymes in the same superfamily can either have native dual activities or evolve to have the activity of other family members. This suggests the possibility that such activity features within a superfamily of proteins might be brought upon in nature through mutations during the evolution process; an outcome that is highly possible for proteins that share the same protein scaffold.

**Figure 1:**
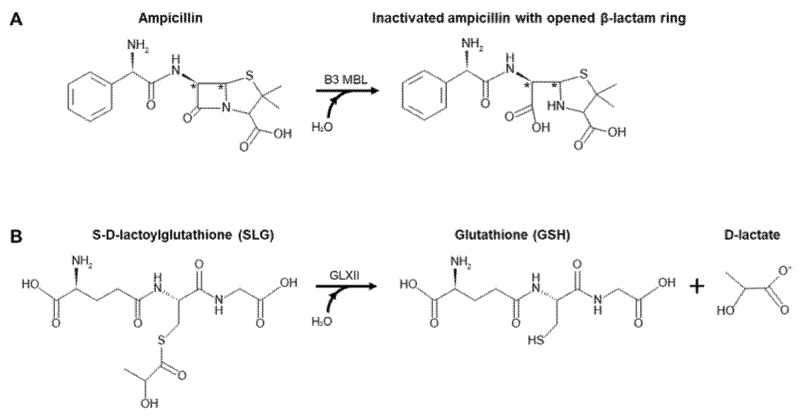
Reaction catalyzed by (**A**) B3 MBL [11] and (**B**) GLXII [13] drawn using MedChem Designer (version 5.5.0.11, Simulations Plus Inc., Lancaster, CA, USA).

Previously, BLEG-1 (formerly termed Bleg1_2437 and Bleg1_2478) from *Bacillus lehensis* G1 was identified as a member of the metallo-hydrolase-like MBL-fold superfamily which exhibited enzymatic activity and predicted structural characteristics similar to the B3 subclass metallo-β-lactamase (MBL) antibiotics-degrading enzyme (E.C. 3.5.2.6) [10]. Interestingly, however, BLEG-1 also showed certain degree of sequence conservation and evolutionary relatedness to GLXII based on BLASTp analysis and postulation by Tan et al. (2017) [10]. Both MBLs and GLXII are members of metallo-hydrolase-like MBL-fold superfamily proteins and they perform different functions. MBLs degrade β-lactam antibiotics by breaking the four-membered β-lactam ring, destructing their core structure and deactivating the antibacterial properties, thus conferring antimicrobial resistance to bacteria (Figure 1A). B3 subclass MBLs are dizinc enzymes and exhibit broad spectrum activity profile towards β-lactam antibiotics, including penicillin, carbapenem and cephalosporins [11]. On the other hand, GLXII (E.C. 3.1.2.6) is one of the key enzymes in the glyoxalase system involved in the detoxification of methylglyoxal, a cytotoxic product of glycolysis and other metabolic activities by converting S-D-lactoylglutathione (SLG) to produce non-toxic D-lactate in the presence of glutathione (GSH) (Figure 1B) [12,13,14].

To date, the possibility of BLEG-1 possessing GLXII activity and the possible reason for its evolutionary divergence from B3 MBLs have not been specifically addressed. The present study reports on the presence of both MBL and GLXII activities in BLEG-1 and the possible reasons that contributed to this aspect through X-ray crystallography and in silico docking analyses of the protein. Comparisons are duly made with its B3 MBL and GLXII counterparts to present a better insight into its substrate recognition and possible binding modes which might have contributed to its dual activity and evolutionary divergence from B3 MBLs.

## 2. Results

### 2.1. Phylogenetic Analysis and Sequence Alignment of BLEG-1

**Figure 2:**
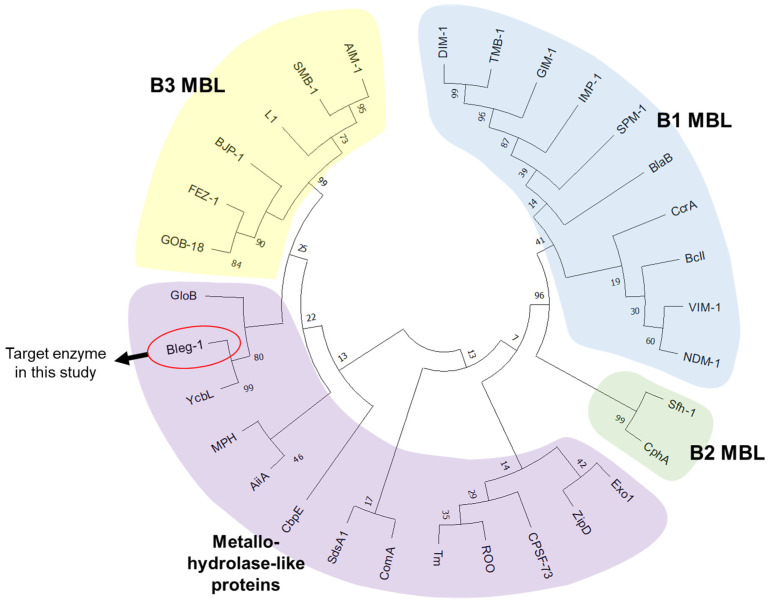
Phylogenetic tree of BLEG-1 with the representative enzymes from each family of the metallo-hydrolase-like MBL-fold protein superfamily, derived using neighbor-joining method. Bootstrap was performed with 100 replicates.

**Figure 3:**
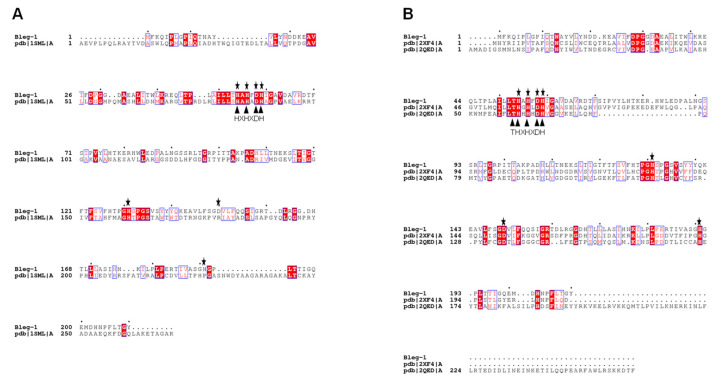
Pairwise sequence alignment of BLEG-1 with (**A**) L1 B3 MBL from *Stenotrophomonas maltophilia* (PDB ID: 1SML, chain A); and multiple sequence alignment of BLEG-1 with (**B**) YcbL, an unusual GLXII from *Salmonella enterica* (PDB ID: 2XF4, chain A) and GloB GLXII from *Salmonella enterica* subsp. enterica serovar Typhimurium str. LT2 (PDB ID: 2QED, chain A). Highly conserved residues are highlighted in red color. Conserved protein motif are marked with black triangles. Zinc coordinating residues in BLEG-1 are marked with stars.

Phylogenetic analysis of BLEG-1 with all three subclasses of MBL and representative members of other metallo-hydrolase-like MBL-fold proteins superfamily revealed that BLEG-1 positioned in the same clade with GLXII and shares a common ancestor as MBL of B3 subclass (Figure 2). This is in agreement to the report by Tan et al. (2017) [10]. In addition, BLEG-1 is evolutionary closest to an unusual member in the GLXII family, which is YcbL from *S. enterica* (PDB ID: 2XF4, chain A). Hence, sequence, structure, and function of BLEG-1 were compared with both B3 MBL, unusual and common GLXIIs to unveil more biochemical and biophysical features of the target enzyme. Sequence alignment of BLEG-1 with the model L1 B3 MBL and closely related GLXIIs showed conservation of His-Xaa-His-Xaa-Asp-His motif and Thr-His-Xaa-His-Xaa-Asp-His motif, which are generally present in most B3 MBLs and GLXIIs respectively (Figure 3).

### 2.2. Steady-State Kinetics Analysis of BLEG-1

**Figure 4:**
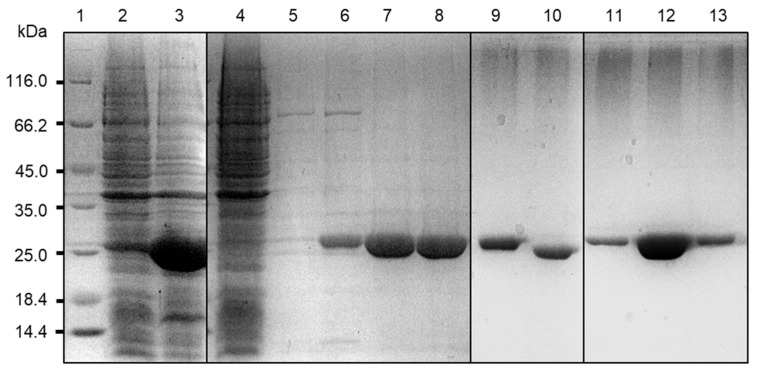
SDS-PAGE (12%) analysis of BLEG-1. Lane 1: unstained protein marker (Thermo Fisher Scientific, Waltham, MA, USA), size range: 14.4-116.0 kDa; lane 2: total cell extract (insoluble fraction); lane 3: total cell extract (soluble fraction); lane 4: HisTrap™ FF flow-through; lane 5 to 8: HisTrap™ FF elution fractions; lane 9: His-tagged BLEG-1; lane 10: thrombin-treated BLEG-1; lane 11 to 13: Q-Sepharose™ FF elution fractions. Molecular weight of BLEG-1 with and without His-tag are 26 and 23 kDa, respectively.

BLEG-1 recombinant proteins, which was overexpressed in the soluble fraction, was purified to homogeneity (Figure 4) to a yield of 0.5 mg/mL after Ni-NTA affinity chromatography. Analysis on the hydrolytic activity of purified BLEG-1 on ampicillin and SLG showed that the enzyme portrayed better affinity (*K_M_*) towards the latter (Table 1). However, BLEG-1 was shown to be able to hydrolyze both substrates with comparable catalytic efficiency (*k_cat_/K_M_*), whereby the efficiency of the reaction towards SLG was only 1.1-fold higher than towards ampicillin, indicating its promiscuity. Most enzymes have a *k_cat_/K_M_* value in the order of 10^5^–10^8^ M^−1^ s^−1^ for their primary or native activity, whereas the magnitude of secondary or promiscuous activity vary over more orders of magnitude and is expected to be lower than the native activity [15]. In the case of BLEG-1, it exhibited similar magnitudes of native and promiscuous activities.

### 2.3. Overall X-ray Crystal Structure of BLEG-1

Distinct, tetragonal shaped BLEG-1 protein crystals with approximate size of 0.6 mm were obtained from optimized formulation containing 17 mg/mL protein in 0.5 M NaI and 30% *w/v* PEG 3350 at 1:1 protein:formulation ratio 20 °C after 14 days of growth. Diffraction of BLEG-1 single protein crystal showed that it has a space group of P4_1_2_1_2 with one molecule in each asymmetric unit, completeness of 98% and R_merge_ of 0.045 (Table 2). Its refined structure has an R_work_ of 18.44% and a corresponding R_free_ of 20.85%. Ramachandran plot analysis on the structure showed that 98.55% of the residues are in favored region and the remaining 1.45% in allowed region (Table 2). The structure of BLEG-1 solved at 1.44 Å resolution was built using molecular replacement (MR) of *S.* *enterica* YcbL: an unusual type II glyoxalase (PDB ID: 2XF4, chain A) [16] as the structural template.

**Figure 5:**
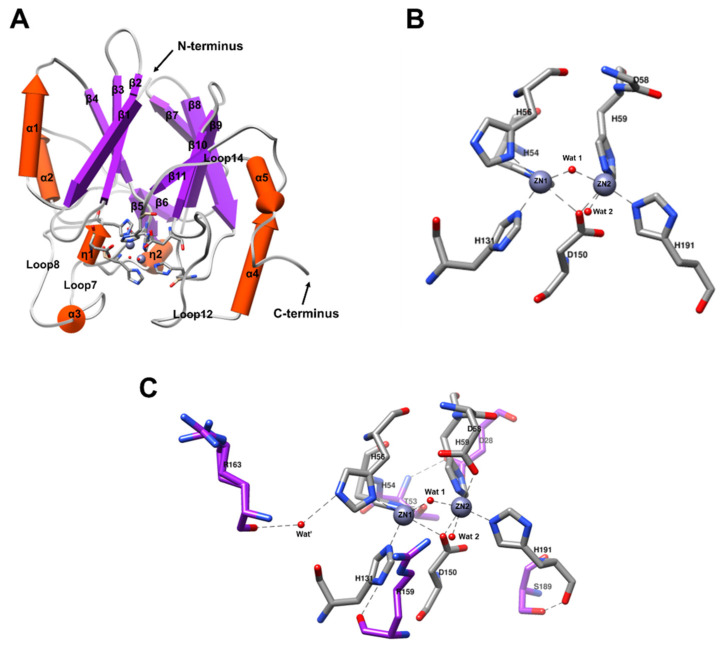
Crystal structure of BLEG-1 solved at 1.44 Å resolution. (**A**) Overall structure of BLEG-1 with marked secondary structure element; (**B**) zinc coordination in active site of BLEG-1 with labelled amino acid residues, zinc ions, and water molecules colored in CPK scheme; (**C**) second shell ligands of the metal binding site of BLEG-1. The metal ligands are labelled and colored in CPK scheme, while carbon atoms of the second shell ligands are illustrated in purple.

Solved 3D structure of BLEG-1 showed a global topology of an αβ/βα sandwich fold, with the active binuclear center located at the interface of N and C-terminal domain (Figure 5A). Each domain consists of an antiparallel β-sheet and two solvent-exposed α-helices. Long loops structures (loop7, loop8, loop12, loop14), two 3_10_-helices (η1, η2), and one short α-helix (α3) sat adjacent to the metal coordination site and form a groove for substrate binding (Figure 5A). At the binuclear center, both zinc ions are coordinated via trigonal bipyramidal geometry in which Zn1 is coordinated by His-54, His-56, and His-131 residues, while Zn2 is coordinated by Asp-58, His-59, and His-191 residues (Figure 5B). This is consistent with its predicted structure reported previously [10]. The zinc ions are separated by a distance of 3.3 Å and are bridged by Asp-150 residue and a water molecule, Wat1 (Figure 5B and Table 3). Another water molecule, Wat2, is interacting with Zn2 at the apical or axial position.

The orientation of histidine residues at the metal binding site is stabilized through second shell interactions. Non-binding N atom in the imidazole ring of histidine residues form subtended hydrogen bonds with side chain or backbone of amino acids, or water molecules in the second coordination shell. In the present structure, Asp-28, Thr-53, Arg-159, Wat’, Arg-163, and Ser-189 act as the second shell ligands of BLEG-1 (Figure 5C, Table 3). They orientate the first shell histidine residues by forming hydrogen bonds with a distance of <3.5 Å. His-54, His-59, and His-191 form hydrogen bonds with the proton acceptor at the side chain of respective second shell amino acid residues, whereas His-131 interacts with the main chain carbonyl oxygen of its second shell ligand, Arg-159. Different from the other residues, His-56 is not orientated by amino acid residues. Instead, it forms hydrogen bond to a water molecule (Wat’) that in turn interacts with Arg-163 at its backbone carbonyl oxygen. Further stabilization of first shell coordination is attained by the interactions between the second shell ligands through the formation of hydrogen bonds, which involved Asp-28 and Thr-53.

### 2.4. Structural Comparison of BLEG-1 with L1 B3 MBL Global Structure and Metal-Center

*S. maltophilia* L1 (PDB ID: 1SML, chain A) [17,18] is a tetrameric MBL from B3 subclass. Each monomer consists of 269 amino acid residues and two zinc ions coordinated by His-84, His-86, His-160, Asp-88, His-89, and His-225 at the active site. L1 is commonly used as a referral model for structural analysis and comparison in MBL-related research [11,19,20,21,22], particularly on the structural and mechanistic features of B3 MBLs.

**Figure 6:**
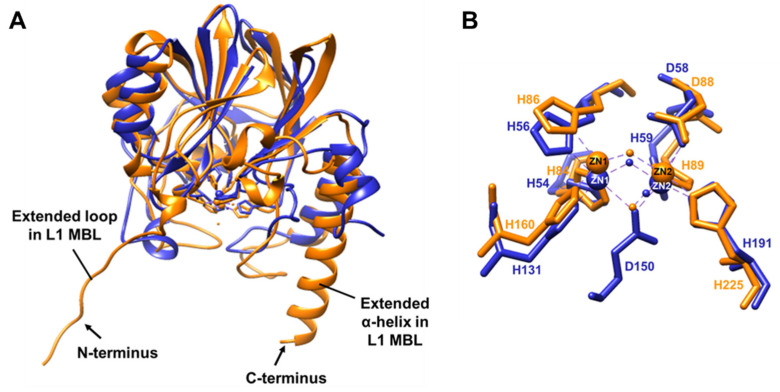
Superimposition of the (**A**) overall protein structure and (**B**) metal coordinating residues of BLEG-1 (blue) with L1 MBL (PDB ID: 1SML, chain A) (orange).

Structural superimposition of BLEG-1 with *S. maltophilia* L1 MBL (PDB ID: 1SML, chain A) [17] gave forth a root-mean-square-deviation (RMSD) of 1.25 Å for 98 equivalent α-carbon, with similar overall protein fold and some significant differences. Comparison of the overall structure of BLEG-1 and L1, showed that L1 consists of an extended N-terminus which forms a mobile segment consisting of two loops (loop1 and loop2) connected by a 3_10_-helix and a β-strand (Ala-1–Ala-27); a feature that is absent in BLEG-1 (Figure 6A). This segment projects outward from the active site and is one of the structures that builds the binding pocket of L1 [17]. Additionally, it has been suggested that this particular extended segment is significant in stabilizing the tetrameric form of L1 MBL through electrostatic and hydrophobic interactions [18]. The C-terminus of L1 on the other hand, has an extended α-helix which is well-conserved in other B3 MBLs such as GOB-18 (PDB ID: 5K0W, chain A) [22], AIM-1 (PDB ID: 4AWY, chain B) [20], SMB-1 (PDB ID: 3VPE, chain A) [21], and FEZ-1 (PDB ID: 1K07, chain A) [23]. This particular long helix at the C-terminus (α6) of L1 interacts with the loop linking α5 and β10 (loop12) by disulphide bond between Cys-256 and Cys-218 (Figure S1A). It was suggested that this interaction may serve to constrain the active site loop in the C-terminal domain of L1 (loop11) [17]. Other than L1, such disulphide interaction was also observed in other B3 MBLs, which are FEZ-1, AIM-1, and SMB-1, which was postulated to be involved in structure stabilization [20,21,23]. The C-terminal extended α-helix was not observed in BLEG-1. Instead, BLEG-1 has a short helix with two turns in place (Figure 6A) and lacks the cysteine residues necessary to form such disulphide bridge observed in L1 MBL.

Despite the above differences, the zinc coordinating residues in the active site of BLEG-1 and L1 are fully conserved (Figure 6B). However, BLEG-1 has an aspartate residue (Asp-150) bridging Zn1 and Zn2 in addition to a “bridging” water (Wat1) molecule. In contrast to this, the zinc ions in L1 were connected solely by a “bridging” water molecule. Based on structural superimposition, Asp-150 in BLEG-1 corresponds to Asp-184 in L1. This residue is not involved in zinc ions interaction at the active site of L1. Instead, it serves as one of the second shell ligands to stabilize zinc conformation [17].

### 2.5. Structural Comparison of BLEG-1 with YcbL and GloB GLXIIs Global Structure and Metal-Center

**Figure 7:**
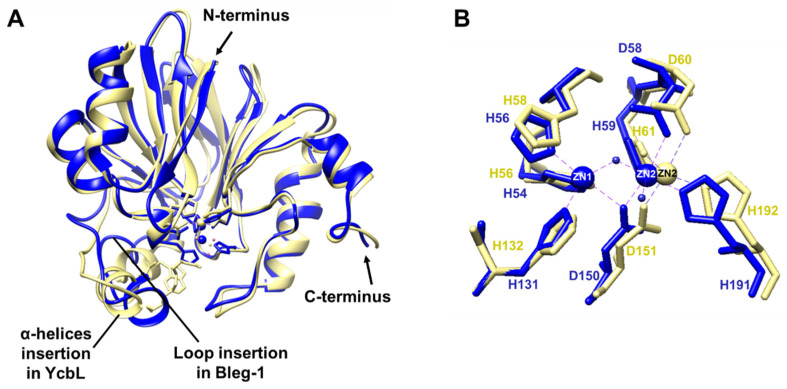
Superimposition of the (**A**) overall protein structure and (**B**) metal coordinating residues of BLEG-1 (blue) with YcbL (PDB ID: 2XF4, chain A) (yellow).

As YcbL GLXII from *S. enterica* (PDB ID: 2XF4) has the second highest identity, well conserved sequence with BLEG-1, and has been biochemically characterized [16], comparison of both protein structures was made. YcbL is a monomeric glyoxalase II comprising of 210 amino acids. Structural superimposition of these proteins yielded an RMSD of 0.89 Å for all 166 equivalent α-carbon, indicating high structural similarities between the enzymes (Figure 7A). Both enzymes lack the C-terminal α-helical domain present in most GLXIIs and have an insertion near the metal center in the N-terminal domain that was not observed in typical GLXIIs. The insertion corresponds to Glu-85-Ala-103 in BLEG-1 (loop7, α3, loop8) and Gln-88-Pro-104 in YcbL (α3) (Figure S1B). Different from BLEG-1, only a single bound Zn ion was found at the active site of YcbL, instead of two which is normally observed in GLXIIs (Figure 7B). Its monozinc ion is coordinated by two histidine and aspartate residues (Asp-60, His-61, Asp-151, and His-192). Close to this site are three histidine residues (His-56, His-58, and His-132) which are structurally aligned with Zn1-coordinating residues in BLEG-1. Although Zn ion was not present in this particular site containing these residues in the crystal structure of YcbL, the projections and orientations of these residues suggest their possible involvement in metal coordination. Additionally, it may be possible that Asp-151 in YcbL is involved in bridging zinc ion with the “apical” water, as seen in BLEG-1, most GLXIIs and B3 MBLs [16,24,25,26].

An additional comparison between the structures of BLEG-1 and GloB, a GLXII from *S. enterica* serovar typhimurium LT2 (PDB ID: 2QED, chain A) [26] was performed as structural search results indicated that GloB is another bacterial GLXII that shared homology with BLEG-1. GloB has an MBL-fold domain and a small α-helices domain at the C-terminus, similar to most GLXIIs [1]. The enzyme exists as a monomer and comprises of 252 amino acid residues and two iron ions coordinated by His-53, His-55, His-110, Asp-57, His-58, and His-165 at the active site. These metal-binding ligands are fully conserved in GLXII family [26].

**Figure 8:**
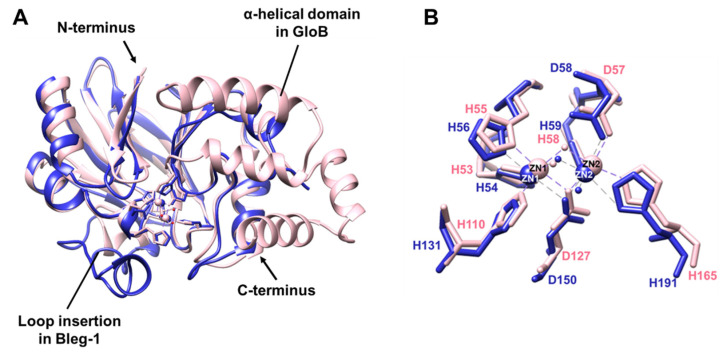
Superimposition of the (**A**) overall protein structure and (**B**) metal coordinating residues of BLEG-1 (blue) with GloB (PDB ID: 2QED, chain A) (pink).

Structural superimposition of BLEG-1 and GloB showed structural similarities between the two structures at certain region, with an RMSD of 0.97 Å for all 131 equivalent α-carbon (Figure 8A). The overall superimposed structures showed that the conformation of the N-terminal domain of both enzymes are somewhat similar, although not as well conserved as YcbL. At the C-terminus however, GloB has an extended domain consisting of 4 α-helices, which is absent in BLEG-1 (Figure 8A). This feature is generally present in all GLXIIs [1,5] and has been reported to be important for substrate binding in GLXIIs [24,27].

At the active site, the metal binding residues of BLEG-1 and GloB are fully conserved. This include the “bridging” residue, Asp-127 in GloB and Asp-150 in BLEG-1 (Figure 8B), both seen coordinating two metal ions. However, the metal ions in GloB are different from those in BLEG-1, which are iron ions as opposed to zinc ions in BLEG-1. This is due to the metal promiscuity of GloB, which can bind to various metal ions such as zinc, iron, and manganese [26]. “Bridging” and “apical” water molecules (Wat1 and Wat2) were also observed in both BLEG-1 and GloB. However, GloB has an extra water molecule (Wat3) interacting with the first metal ion (Figure 8B).

### 2.6. Active Site Configuration of BLEG-1, L1 B3 MBL, YcbL, and GloB GLXIIs

**Figure 9:**
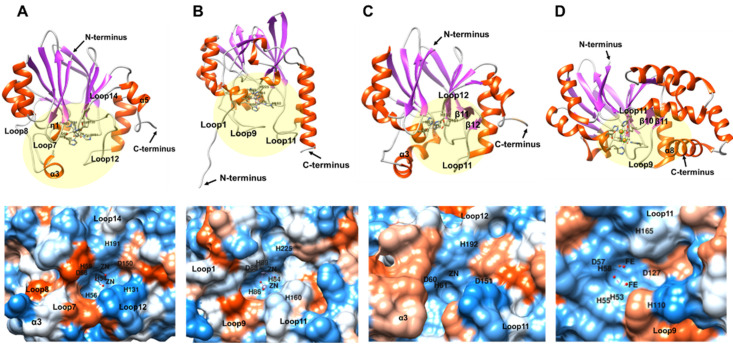
Crystal structures illustrated in ribbon view with active site indicated by yellow circle (**top**) and surface depiction of active-site groove colored in hydrophobicity scale (blue for the most hydrophilic, white to red for the most hydrophobic) (**bottom**) of (**A**) BLEG-1; (**B**) L1 MBL (PDB ID: 1SML, chain A); (**C**) YcbL (PDB ID: 2XF4, chain A); and (**D**) GloB GLXII (PDB ID: 2QED, chain A).

The active sites of both BLEG-1 and L1 mainly consisted of loops (Figure 9A,B, top). Compared to L1, the binding pocket of BLEG-1 has shorter loops of approximately 10–20 residues in length. They are loop7 (Arg-81–Gly-91), loop8 (Gly-97–Asp-107), loop12 (Asp-150–Asp-166), and loop14 (Gly-190–Thr-196). Loop7 and 8 are connected by an extra helical insert (α3). In L1, the loops at the active site are longer, composing of approximately 20–30 residues. They are loop1+η1+loop2 (Ala-1–Ala-27), loop9 (Arg-116–Glu-143), and loop11 (Asp-184–His-201). Structural superimposition showed that loop7+α3+loop8 of BLEG-1 and loop9 of L1 are aligned, demonstrating analogous packing at their active site at the N-terminal domain. These loops flanking the N-terminal area of the active sites of both enzymes provided a deep cavity to this region (Figure 9A,B, bottom). At the C-terminal region of the active site, the extended α-helix structures observed in L1 interacted with loop11 by electrostatic and hydrophobic interactions (Figure 9B, top), making the loop protrude away from the metal center and forming a deep cavity with other loops at the active site (Figure 9B, bottom). In BLEG-1, in place of this extended α-helix structure is a two-turn short helix (α5) instead (Figure 9A, top). The short α-helix structure (α5) in BLEG-1 does not exert much constrain to the active site loop (loop12) and caused it to be packed closer to the metal center of BLEG-1, giving forth a shallower cavity in the C-terminal region of BLEG-1 active site (Figure 9A, bottom) relative to L1 (Figure 9B, bottom).

Unlike BLEG-1 and L1 MBL, the active sites of YcbL and GloB are mainly comprised of shorter loops and turns of less than 10 amino acid residues (Figure 9C,D, top). These short loops contributed to wider and shallower active site grooves in both GLXIIs (Figure 9C,D, bottom). The flanking loop (loop7+α3+loop8) observed at the N-terminal area of the active site of BLEG-1 is absent in GloB. However, this additional feature was observed in YcbL, albeit being folded into an α-helical structure, suggesting its higher rigidity. In addition to this, structural alignment was observed at the C-terminal region of the active sites between loop12 of BLEG-1 and β10+β11+loop11 of YcbL and β11+β12+loop9 of GloB, resulting similar active site topology in this region.

### 2.7. In Silico Substrate Binding Analyses of BLEG-1, L1 MBL, YcbL GLXII, and GloB GLXII

#### 2.7.1. Docking of BLEG-1 and L1 MBL with Ampicillin

**Figure 10:**
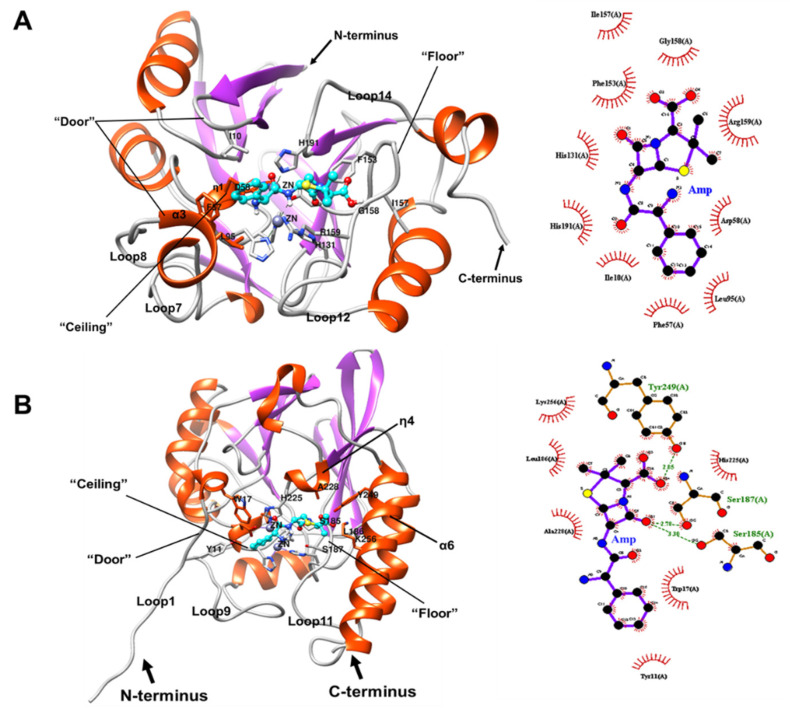
3D complex structure (**left**) and 2D interaction diagram (**right**) of (**A**) BLEG-1-ampicillin and (**B**) L1 MBL-ampicillin. In 3D complex structure, ampicillin molecule is colored in cyan. In the 2D interaction diagram, red spoked arc represents hydrophobic interaction or non-bonded contact, green dash indicates hydrogen bond, and purple solid line represents ligand bond.

Docked structures of ampicillin with crystal structures of BLEG-1 and L1 (PDB ID: 1SML, chain A) recorded similar values of the scoring function. Figure 10A illustrates the binding of ampicillin in BLEG-1 binding pocket by hydrophobic interactions that were constituted by 10 amino acid residues within the distance of 5 Å to ampicillin. These residues include the zinc-coordinating residues: Asp-58, His-131, His-191, residues in loop1: Ile-10, η1: Phe-57, α3: Leu-95, and loop12: Phe-153, Ile-157, Gly-158, and Arg-159, showing partial agreement to the report by Selvaraju et al. (2020) [28]. Our results showed that the binding mode of ampicillin in BLEG-1 and L1 are similar in which the aromatic side chain of ampicillin (i.e., 2-amino-2-phenylacetamido group) and penam ring are mainly accommodated by the active site loops in the N- and C-terminal domain respectively (Figure 10A,B). Notably, ampicillin was positioned in similar orientation in the docked models of BLEG-1 and L1 as the hydrolyzed penicillin in L1-penicillin complex structure (PDB ID: 6U0Z, chain A) [29]. The aromatic side chain of penicillin in the complexed L1 structure was not well defined as it was proposed to be caused by disorder following exposure of the phenyl group to solvent as it orients upward [29]. Such substrate orientation in L1-penicillin complex was also observed in our docked models. In BLEG-1, Ile-10 (loop1), Phe-57, Asp-58 (η1), and Leu-95 (α3) formed a hydrophobic pocket in the N-terminal region that binds to the aromatic side chain of ampicillin (Figure 10A). This corresponds to the accommodation of ampicillin in L1 in our docking analysis (Figure 10B), as well as in the previous report by Ullah et al. (1998) [17] in which the non-polar amino acids in the N-terminal domain provide a hydrophobic environment for the interactions of β-lactam that bear a hydrophobic side chain. On the other hand, penam ring of ampicillin is interacted by the active site loop in the C-terminal domain of two enzymes, i.e., loop12 in BLEG-1 and loop11 in L1, which are structurally aligned. Although these two loops are packed into a different configuration in the C-terminal region of BLEG-1 and L1, their role in ampicillin accommodation is similar. In addition, docking results showed that the β-lactam ring of ampicillin is located approximately 5 Å to the bridging water and apical water in BLEG-1 and L1, suggesting possible nucleophilic attack of the β-lactam ring by water molecule at dynamic state. The distance can be possibly less in actual situation, and this would require further investigations through crystallization of BLEG-1-substrate complex, along with molecular dynamics simulations that would take into the account the dynamics or motions of the system involved. The main difference in ampicillin binding in BLEG-1 and L1 is that the ampicillin is bound solely by hydrophobic interactions in BLEG-1, whereas in L1, formation of hydrogen bonds between Ser-185 (loop11), Ser-187 (loop11), and Tyr-249 (α6) with the ampicillin was observed (Figure 10B). Ser-185 and Ser-187 in L1 formed hydrogen bond with the carbonyl oxygen of penam ring in ampicillin by the hydroxyl group in their side chain. These two serine residues in L1 are not conserved in BLEG-1 and are structurally aligned to Val-151 and Phe-153, respectively, which constituted of hydrophobic side chain and could not form hydrogen bond with ampicillin. Another hydrogen bond between the hydroxyl group of Tyr-249 in L1 and carboxylate group of penam ring in ampicillin (Figure 10B), was not observed in BLEG-1 as the residue was not conserved.

In addition to the key residues involved in substrate binding, it has also been proposed that the flexible loop structures nearby the active site of MBLs allow accommodation of various β-lactams, whereby the length of loops contributes to their broad substrate profile [11,30]. They play important structural and functional roles by forming the “door”, “ceiling”, and “floor” architectures in B1 MBLs such as NDM-1, IMP-1, and VIM-2 [31] and some B3 MBLs such as L1, AIM-1, and SMB-1 [17,20,32]. In this study, the L1-ampicillin docked model showed that the “ceiling and floor” architectures in L1 active site are possibly composed of a short loop at Zn1 coordination site (Ser-83–His-89) and a short segment in loop11 (Asp-184–Gly-190), respectively (Figure 10B, left), as similarly reported previously by Ullah et al., 1998 [17]. The hydrophobic region of the extended N-terminus (Tyr-12–Leu-18) in L1 is predicted to serve as a “door” that governs the entrance of β-lactams; and covers the binding cavity upon substrate binding [31]. These architectures are also observed at the active site of BLEG-1-ampicillin docked structure and showed agreement to the report by Tan et al. (2017) [10], whereby its “ceiling” is most probably represented by η1 (Thr-53–His-59) and its “floor” is represented by loop12 (Asp-150–Thr-160) (Figure 10A, left). Although the equivalent “doorkeeper” sequence in L1 is not observed in BLEG-1, it is believed that the short loop segment connecting β1 and β2 (Gly-8–Gln-11) and the α3 short helical insert (Ser-92–Thr-96) act as the “door” in BLEG-1 (Figure 10A, left). These two structures are rich in hydrophobic amino acids and control the entrance of β-lactams by interacting with the aromatic side chain of ampicillin. This corresponds to the postulation that the “doorkeeper” structure is mostly comprised of hydrophobic residues in L1 and B1 MBLs (i.e., IMP-1 and NDM-1) [31].

#### 2.7.2. Docking of BLEG-1, YcbL, and GloB GLXII with S-D-Lactoylglutathione (SLG)

**Figure 11:**
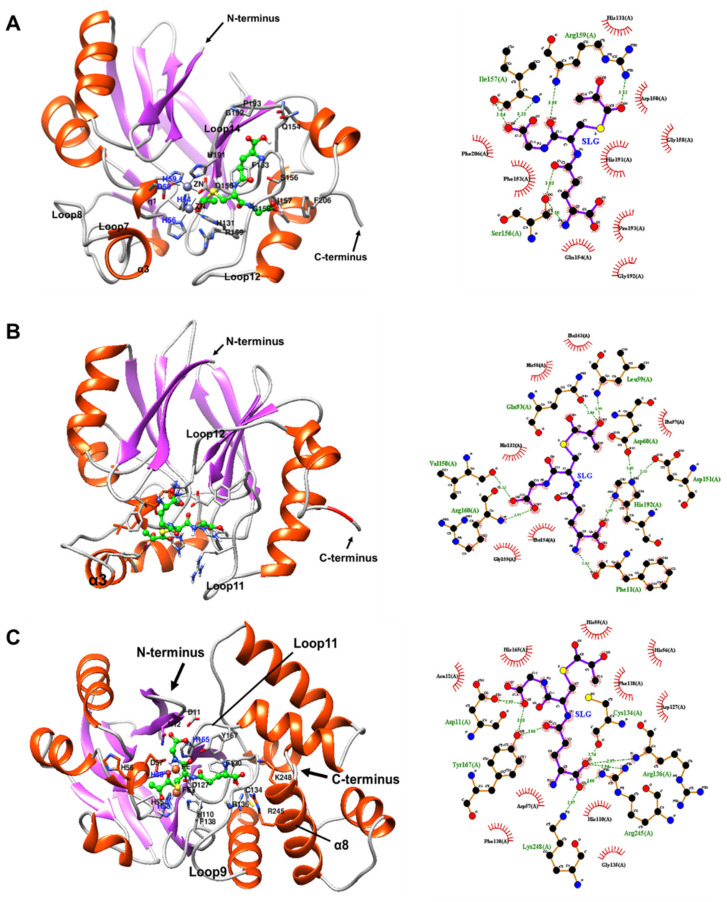
3D complex structure (**left**) and 2D interaction diagram (**right**) of (**A**) BLEG-1-SLG, (**B**) YcbL-SLG, and (**C**) GloB-SLG. In 3D complex structure, SLG molecule is colored in green. In the 2D interaction diagram, red spoked arc represents hydrophobic interaction or non-bonded contact, green dash indicates hydrogen bond, and purple solid line represents ligand bond.

To identify S-D-lactoylglutathione (SLG) binding site in BLEG-1 and GLXII, docking of SLG molecule to the crystal structures of BLEG-1, YcbL (PDB ID: 2XF4, chain A), and GloB (PDB ID: 2QED, chain A) were conducted. The SLG complex structures of BLEG-1, YcbL, and GloB gave forth binding scores of −5.6, −5.3, and −6.5, respectively. BLEG-1 binds SLG in the active site by 12 residues through hydrophobic interactions and hydrogen bonding. These residues include the metal-binding ligand His-131, residues in loop12: Asp-150, Phe-153, Gln-154, Ser-156, Ile-157, Gly-158, Arg-159; loop14: His-191, Gly-192, Pro-193; as well as Phe-206 (Figure 11A). In general, SLG accommodation in BLEG-1, YcbL, and GloB are mainly governed by the loop structures in the C-terminal domain (Figure 11 and Table 4). Loop12 and loop14 of BLEG-1 interacts with the Gly- and Glu-moieties of SLG molecule, respectively. This shows similarity to the SLG binding mode in YcbL in which the structurally aligned loop segments, i.e., loop11 and loop12 in YcbL also establish interactions with the Gly- and Glu-moieties of SLG respectively (Table 4). However, in GloB, the positioning of SLG in the active site is slightly different; in which the corresponding loop structures in the C-terminal domain of GloB, i.e., loop9 and loop11 bind to Glu- and Gly-moieties respectively (Table 4). Notably, Glu-moiety of SLG is further stabilized in GloB by Arg-245 and Lys-248 (α8) in its α-helical domain by hydrogen bonding (Figure 11C). Arg-245 and Lys-248 had been postulated to be involved in substrate accommodation of GloB [14,26], and our docking analysis showed agreement to their proposition. Based on our observation, α8 and loop9 of GloB are found in the same vicinity, therefore it is possible for α8 to establish contacts with SLG molecule during substrate binding. Interaction of the Cys-moiety of SLG in BLEG-1, YcbL and GloB is mainly through the metal-coordinating residues and their neighboring residues, i.e., BLEG-1: His-131 and Asp-150; YcbL: His-58, Leu-59 and His-60; GloB: His-55, His-56 and Asp-127. As the thiol group of cysteine permits metal binding [33], recognition of Cys-moiety of SLG in BLEG-1, YcbL and GloB might be driven by the metal ion(s) and stabilized by the residues in the proximity to metal center [14]. In YcbL and GloB, a phenylalanine residue in the active site loop of their C-terminal domain, which projects toward the metal center, i.e Phe-163 (loop11) in YcbL; and Phe-138 (loop9) in GloB, also showed interactions with the Cys-moiety of SLG (Figure 11B,C). This phenylalanine residue corresponds to Tyr-145 in human GLXII that demonstrated substrate interaction [24], but was proposed that it might not be involved in SLG accommodation in both YcbL and GloB [16,26]. Our analysis however, showed that Phe-163 (in YcbL) and Phe-138 (in GloB) bind SLG molecule by hydrophobic interactions. In BLEG-1, this residue is not conserved and is substituted by Leu-162 (loop12). Unlike the phenylalanine residue in YcbL and GloB, Leu-162 did not interact with SLG molecule in BLEG-1, possibly due to their distinct side chain. The bulky aromatic side chain of phenylalanine may impose stronger interactions to SLG molecule than leucine, which has an aliphatic side chain [34]. Additionally, Gln-93 and Phe-97 in the α-helix insert (α3) of YcbL also showed interactions with the Cys-moiety of SLG by hydrophobic interactions and hydrogen bond respectively (Figure 11B). These two residues correspond to Ser-92 and Thr-96 in BLEG-1, which have short side chain and thus might not be possible to form interaction with SLG as Gln-93 and Phe-97 in YcbL.

## 3. Discussion

In addition to the report by Tan et al. (2017) [10] that BLEG-1 demonstrates biochemical and structural characteristics of B3 MBL, our results indicate the possibility that the enzyme may also possess the characteristics of GLXII.

YcbL has been designated as GLXII-2, an isozyme of GLXII that catalyze the hydrolysis of SLG at lower rate [35]. Similar to YcbL, the *k_cat_/K_M_* of BLEG-1 is lower than that of typical GLXIIs. The comparable catalytic efficiency of BLEG-1 towards both ampicillin and SLG, however, sets it apart from YcbL which lacked MBL activity. It is possible that BLEG-1 exhibits native and promiscuous activities at lower rate compared to other B3 MBLs and GLXIIs (Table S2) as the introduction of a new function to an enzyme during evolution could be accompanied by large decrease in its primary activity [15].

Analyses on the global structure of BLEG-1, L1 B3 MBL, YcbL and GloB GLXIIs revealed that the enzyme showed higher resemblance to GLXII, particularly to YcbL, a GLXII-2. Yet, the active site architecture of BLEG-1 resembled that of L1 B3 MBL, which allowed the accommodation of β-lactam other than SLG. This feature may have contributed to its enzyme promiscuity. Our docking analyses of BLEG-1 with ampicillin and SLG revealed that the β-lactam ring of ampicillin and Cys-moiety of SLG are found in the vicinity of metal ligands, indicating that the accommodation of both substrates might be governed by the metal ions at the active site. This corresponds to the postulations that the metal ligands in the active site are significant in establishing electrostatic interactions with ampicillin and SLG [24,36]. SLG binds to the exposed and shallow binding cleft at the C-terminal domain of BLEG-1 which possess similar conformation as the active site of GLXIIs. Parallel to the report by Stamp et al. (2010) [16], our docking analysis of BLEG-1 with SLG indicated that binding of SLG is possible in the absence of the C-terminal α-helical domain. However, it should not be denied that this domain is significant for GLXII activity. Mutational and mechanistic studies on *Arabidopsis* GLXII had proved that mutation of Arg-248 that interacted with SLG molecule in the α-helical domain resulted in 10-fold reduction in catalytic activity [37]. This explains the comparatively low catalytic efficiency of BLEG-1 and YcbL towards SLG in the GLXII family. Additionally, SLG binding in BLEG-1 seemed to be hindered in the N-terminal domain due to the presence of a gorge-forming loop structure (loop7+α3+loop8). This flanking loop structure (loop7+α3+loop8) which is of high hydrophobicity, however, showed significance in binding of ampicillin in BLEG-1. It associates with the loops in C-terminal region and stabilized ampicillin in the active site through hydrophobic interactions. This observation suggests that the extended N-terminal segment in B3 MBLs is not mandatory for accommodation of β-lactam [18]. Instead, the proximity of hydrophobic surfaces in the active site groove is more important for this purpose, by lodging the aromatic side chain of the ampicillin through hydrophobic interactions. This is in agreement to the previous observation reported by Ullah et al. (1998) [17]. Nonetheless, loop flexibility and dynamics in MBLs had proved to be important for β-lactam binding and their broad substrate spectrum [38]. We, therefore, postulate that the high hydrophobicity in the binding pocket and the insertion of active-site loop (loop7+α3+loop8) in the N-terminal domain of BLEG-1 has contributed to the configuration of a binding cavity that allowed ampicillin to bind. In contrast to this, YcbL which has a similar sequence insertion that is similarly hydrophobic as BLEG-1, has an α-helical structure in place of the loop observed in BLEG-1 and this did not allow accommodation of ampicillin in the protein. This could explain the absence of MBL activity in YcbL [16], due to the low plasticity of its binding pocket.

In general, based on our results in this study, we suggest that BLEG-1 could have evolved from GLXII and might be an intermediate in the natural divergence path of B3 MBLs as follows: Unknown origin → GLXII → BLEG-1 → B3 MBLs. To date, there are reports postulating that MBLs are descended from other members in the metallo-hydrolase-like MBL-fold protein superfamily for their stable protein scaffold, i.e., MBL-fold, which has been repetitively used during evolution lead to the emergence of catalytically diverse enzymes in the superfamily. This diversity in function is contributed by altering sequence and length of active site loops of the enzymes to accommodate various substrates. It has also been suggested that certain superfamily members can be easily evolved into MBL due to their exposure to environmental antibiotics [5,8,39,40]. Therefore, it is possible that BLEG-1, initially descended from GLXII, adopted active site configuration that allows the accommodation of β-lactam antibiotics during evolution, as a consequence of antibiotic stress. Upon the completion of natural divergence path, BLEG-1 may have evolved into B3 MBL with partial abolishment of GLXII activity, which may have been brought about by more mutations on the active site loops especially on the C-terminal domain [15], causing it to lose the structural feature of GLXII at this particular domain. It would be of interest for future investigations to perform mutagenesis of the active site loop (loop7+α3+loop8) in the N-terminal domain of BLEG-1 as well as the C-terminal domain, respectively; and characterize the effect on its activity and flexibility in addition to crystallization of BLEG-1-substrate complex to gain better insights into its mechanistic function.

## 4. Materials and Methods

### 4.1. Bacterial Strains and Plasmids

*Escherichia coli* BL21(DE3) containing the previously constructed recombinant plasmid pET28(b)::*bleg-1* carrying the codon-optimized *bleg-1* open-reading-frame (Tan Soo Huei and Yahaya M. Normi, 2018, unpublished data) [41] was used as the host for heterologous production of BLEG-1 recombinant protein.

### 4.2. Phylogenetic Analysis of BLEG-1

Phylogenetic analysis of BLEG-1 was performed with the representative enzymes from each family of the metallo-hydrolase-like MBL-fold protein superfamily reported by Bebrone (2007) [5], Daiyasu et al. (2001) [1], and Palzkill (2013) [11] as listed in Table S1. By using the MEGA software (version 10.1.6, Pennsylvania State University, Pennsylvania, USA) [42], the amino acid sequences of BLEG-1 and representative enzymes were aligned using ClustalW [43] and subsequently subjected to phylogenetic tree construction using neighbor-joining algorithm with 100 bootstrap replications.

### 4.3. Sequence Alignment of BLEG-1

By using the ClustalW algorithm in the MEGA software (version 10.1.6, Pennsylvania State University, Pennsylvania, USA) [42], the amino acid sequence of BLEG-1 (UniProt accession number: A0A060M4R1) was subjected to pairwise sequence alignment and multiple sequence alignment with the selected referral models from the B3 MBL and GLXII family, respectively, to identify sequence conservation. The sequence alignment results were visualized using ESPript (version 3.0.8, CNRS UMR5086 Lyon University, Lyon, France) [44].

### 4.4. Overexpression and Purification of BLEG-1

Following the method by Tan et al. (2017) [10] with slight modifications, *E. coli* BL21(DE3) cells harboring pET-28b(+)::*Bleg-1* recombinant plasmid were cultivated in 120 mL Luria-Bertani (LB) medium containing 50 μg/mL of kanamycin at 37 °C for 18 h, with agitation at a speed of 200 rpm. 120 mL of this culture was used to inoculate a total of 3 L LB medium containing 50 μg/mL of kanamycin and 0.1 mM ZnSO_4_. Cells were cultivated at 37 °C with agitation until the optical density of this culture at 600 nm (OD_600_) reached 0.6. Then, 0.1 mM of isopropyl-β-D-thiogalactopyranoside (IPTG) was added into the culture and further cultivated at 20 °C for 20 h. The cells were then harvested by centrifugation at 9000× *g*, 4 °C for 20 min.

The cell pellet was lysed in 50 mL of buffer 1 (20 mM sodium phosphate buffer, 500 mM NaCl, 50 mM imidazole, 2 mM MgSO_4_•7H_2_O, pH 7.4) by sonication at 40% amplitude, 15 s ON and 15 s OFF pulses for 12 min on ice, followed by centrifugation at 9000× *g*, 4 °C for 20 min. The supernatant was collected and loaded into 5 mL pre-packed HisTrap^TM^ FF column (GE Healthcare, Chicago, IL, USA), which was pre-equilibrated with buffer 1, at flow rate of 1.0 mL/min. The column was washed with 20 column volume (CV) of buffer 1 and the bound protein was eluted by gradient elution using 20 CV of buffer 2 (20 mM sodium phosphate buffer, 500 mM NaCl, 500 mM imidazole, pH 7.4), at the same flow rate. Flow-through and elution fractions were analyzed using SDS-PAGE (12%). The eluted protein was subjected to dialysis in 4 L of buffer 3 (20 mM sodium phosphate buffer, 20 mM NaCl, pH 7.4) at 4 °C for 18 h using SnakeSkin Dialysis Tubing, 10,000 MWCO (Thermo Fisher Scientific, Waltham, MA, USA). His-tag cleavage was then performed by adding 1 U/µL of thrombin (Merck, Darmstadt, Germany) per mg/mL of His-tagged fusion protein and incubated at 25 °C for 16 h. His-tag and thrombin were subsequently removed via anion exchange chromatography, by loading thrombin-treated protein sample into a 5 mL Q-Sepharose™ FF column (GE Healthcare, Chicago, Illinois, USA), which was pre-equilibrated with dialysis buffer (buffer 3) at flow rate of 1 mL/min. The bound protein was washed with 12 CV of buffer 3 and eluted by gradient elution with 20 CV of buffer 4 (20 mM sodium phosphate buffer, 500 mM NaCl, pH 7.4). The collected fractions were analyzed using SDS-PAGE (12%), pooled, and dialyzed using the same dialysis tubing as mentioned above in 4 L buffer 5 (20 mM sodium phosphate buffer, 100 mM NaCl, 0.1 mM ZnSO_4_, 5% glycerol, pH 7.4) at 4 °C for 18 h. Purified protein was concentrated via centrifugation using Vivaspin 6 centrifugal concentrator (Sartorius, Göttingen, Germany) with MWCO of 10 kDa. Concentrations of protein samples were determined via Bradford method [45], using Bradford Reagent (Sigma-aldrich, St. Louis, MI, USA) with bovine serum albumin as standard and measured at 595 nm.

### 4.5. Steady-State Kinetic Analysis of BLEG-1

Kinetic assays were conducted in a 1 mL ES Quartz cell (E-Chrom Tech, Taipei, Taiwan) with a UV-1800 UV/Visible Scanning Spectrophotometer (Shimadzu, Kyoto, Japan) using purified BLEG-1 protein. MBL and GLXII activities of BLEG-1 were tested using ampicillin and S-D-lactoylglutathione (SLG) as respective substrates. Hydrolysis of β-lactam was performed based on the method described by Tan et al. (2017) [10] with slight modifications. The assay was carried out at 30 °C in 1 mL volume of 20 mM sodium phosphate buffer, pH 7 containing 1 µM of purified BLEG-1 protein, 20 µg/mL bovine serum albumin, 100 µM ZnSO_4_, and ampicillin at concentrations ranging from 0–200 µM. The changes in molar absorptivity (∆ε) of ampicillin, ∆ε_235_ = –800 M^−1^ cm^−1^ was used to quantitate the hydrolyzed ampicillin, and the changes in absorbance (∆A) versus time were measured for a period of 210 s.

Hydrolysis of SLG by BLEG-1 was investigated based on the method outlined by Racker (1951) [46] with modifications. The assay was carried out at 37 °C in 1 mL volume of 20 mM sodium phosphate buffer, pH 7 containing 1 µM of purified BLEG-1 protein, 20 µg/mL bovine serum albumin, 100 µM ZnSO_4_, and SLG at concentrations ranging from 0–200 µM. The changes in molar absorptivity (∆ε) of SLG, ∆ε_240_ = 3370 M^−1^ cm^−1^ was used to quantitate the unhydrolyzed SLG, and the changes in absorbance (∆A) versus time were measured for a period of 210 s. All assays were carried out in triplicates. The enzyme activity was defined as the μmol of substrate being hydrolyzed per minute, µmol min^−1^. Steady-state kinetics constants, *K_M_* and *k_cat_*, were calculated by fitting initial velocity versus substrate concentration to the Lineweaver–Burk equation [47].

### 4.6. Protein Crystallization, Diffraction, and Data Collection

Crystal pre-screening of BLEG-1 was carried out using the sitting drop vapor diffusion method at 4 °C with the following commercial screening kits: Crystal Screen 1™ and Crystal Screen 2™ (Hampton Research, Aliso Viejo, CA, USA), Index Screen™ (Hampton Research, Aliso Viejo, CA, USA), PEG/Ion Screen 1™ and PEG/Ion Screen 2^TM^ (Hampton Research, Aliso Viejo, CA, USA), and JCSG plus™ (Molecular Dimensions, Sheffield, UK). Purified BLEG-1 at concentrations of 3 and 17 mg/mL, respectively, was used at 1:1 and 2:1 protein:formulation ratios to identify the suitable protein concentration and formulation for BLEG-1 crystal growth. Further optimization was performed by varying PEG3350 concentrations: 10%, 20%, and 30% (*w*/*v*); and NaI concentrations: 0.1, 0.2, 0.3, 0.4, and 0.5 M using the formulation that formed protein crystals. Incubation temperature for crystal growth was also screened at 4 °C and 20 °C, respectively. Single protein crystal obtained at the optimum conditions was selected and diffracted with an in-house X-ray diffractometer (Rigaku Micromax-007 generator) equipped with a CCD MSC PAD200sk detector at Malaysian Genome Institute, Selangor, Malaysia, at 100 K and wavelength of 1.54 Å. The diffraction data was processed and scaled with Denzo and SCALEPACK in the HKL 3000R software (version v714n, HKL Research Inc., Charlottesville, VA, USA) [48], respectively.

### 4.7. Structure Determination, Refinement, and Validation

Molecular replacement (MR) was used to solve the atomic structure of BLEG-1 via the Phaser—MR program in Phenix software (version 1.16-3549, Lawrence Berkeley Laboratory, Berkeley, California, USA) [49,50], before being modelled and rebuilt manually using the COOT software (version 0.8.9.2, MRC LMB, Cambridge, UK) [51]. Structural template for MR was retrieved based on the BLASTp search against Protein Data Bank (PDB). Automated restraint refinement was performed using the phenix.refine program in the Phenix software (version 1.16-3549, Lawrence Berkeley Laboratory, Berkeley, CA, USA) [50,52] and was subsequently validated using Molprobity [53] associated with the Phenix software (version 1.16-3549, Lawrence Berkeley Laboratory, Berkeley, CA, USA). Structural analyses and superimposition of BLEG-1 with selected MBL and GLXII enzymes were performed using the UCSF Chimera software (version 1.13.1, RBVI UCSF, San Francisco, CA, USA) [54].

### 4.8. Docking Analyses of BLEG-1 and Selected MBL and GLXII

3D conformer of ampicillin and SLG (the substrates) were retrieved from PubChem database (https://pubchem.ncbi.nlm.nih.gov/, accessed on 6 April 2020) and used for docking onto BLEG-1, and reference B3 MBL, L1 from *Stenotrophomonas maltophilia* (PDB ID: 1SML, chain A), and selected GLXII enzymes YcbL from *Salmonella enterica* (PDB ID: 2XF4, chain A) and GloB from *Salmonella enterica* subsp. enterica serovar Typhimurium str. LT2 (PDB ID: 2QED, chain A) using the AutoDock software (version 4.2.6, The Scripps Research Institute, La Jolla, California, USA) [55]. A grid box of 40 × 40 × 40 Å centered at the active site of BLEG-1 and L1 B3 MBL enzyme with grid-point spacing of 0.464 Å and 0.375 Å, respectively, was used for docking of ampicillin; while grid-point spacing of 0.469 Å, 0.375 Å, and 0.461 Å was used for docking of SLG onto BLEG-1, YcbL, and GloB GLXIIs, respectively. Substrate binding residues of BLEG-1 were predicted based on the conservation of amino acid observed in respective structural pairwise alignment with the selected MBL and GLXII enzymes, prior to defining its grid-point spacing. Low binding scores and appropriateness of pose were used as indicators to select substrate binding pose. Interactions of substrates in enzyme structures were analyzed using the LIGPLOT+ software (v.2.1, EMBL-EBI, Cambridge, UK) [56], followed by the UCSF Chimera software (version 1.13.1, RBVI UCSF, San Francisco, CA, USA) [54].

## 5. Conclusions

In this study, we demonstrated that BLEG-1 possessed dual MBL and GLXII activities with similar catalytic efficiencies. Based on the conservation of Thr-His-Xaa-His-Xaa-Asp-His protein motif and “bridging” residue which are strongly conserved in the GLXII family, as well as the phylogenetic relatedness and higher similarity in the overall protein structure between BLEG-1 and GLXII, we suggest BLEG-1 could have evolved from GLXII. The crystal structure of BLEG-1 solved at 1.44 Å resolution unveiled structural features which may have contributed to its promiscuity and evolutionary divergence. The insertion of active-site loop in the N-terminal domain of BLEG-1 active site formed a configuration similar to B3 MBL binding cavity which eventually allowed the accommodation of β-lactams. This study has given new insights into the structural variations of GLXII and B3 MBL, thus delineating their promiscuity and evolutionary paths from a structural perspective. The wide distribution of metallo-hydrolase-like MBL-fold proteins and the evolvability of mechanistically distinct superfamily members into MBLs underline a “hidden” threat of such bacterial adaptation which could have arose from antibiotic stress.

## Figures and Tables

**Table 1:** Kinetics parameters of BLEG-1 towards ampicillin and SLG.

	With Ampicillin	With SLG
*V_max_* (mM s^−1^)	(4.20 ± 0.16) × 10^−5^	(3.55 ± 0.26) × 10^−5^
*K_M_* (mM)	(7.27 ± 0.51) × 10^−2^	(5.50 ± 0.12) × 10^−2^
*k_cat_* (s^−1^)	(4.20 ± 0.16) × 10^−2^	(3.55 ± 0.26) × 10^−2^
*k_cat_/K_M_* (mM^−1^ s^−1^)	(5.78 ± 0.19) × 10^−1^	(6.47 ± 0.77) × 10^−1^

**Table 2:** Statistics of BLEG-1 crystallographic structure determination.

Data Collection Statistics	
Data collection site	Malaysia Genome Institute
Detector	CCD MSC PAD200sk
Wavelength (Å)	1.5418
Data resolution (Å)	50.00–1.44 (1.46–1.44)
Space group	P4_1_2_1_2
Unit cell dimensions (a, b, c) (Å)	55.36, 55.36, 143.09
Total number of reflections	205685
Number of unique reflections	40528
Multiplicity	5.1 (3.1)
Completeness (%)	98.0 (97.9)
R_merge_	0.045 (0.254)
<I/σ(I)>	28.5 (4.0)
**Refinement Statistics**	
Resolution range (Å)	30.26–1.44 (1.49–1.44)
No: residues/water/ion/ligand	209/245/11/2
R-factor (R_work_/R_free_) (%)	18.44/20.85
Rms bond length deviation (Å)	0.005
Rms bond angle deviation (°)	0.804
Ramachandran angles (favored/allowed/disallowed) (%)	98.55/1.45/0

**Table 3:** Coordination of zinc ions at active site of BLEG-1.

Metal ion	Distance (Å)	First Shell Ligand		Second Shell Ligand
		Involved in Metal Coordination	Involved in Second Shell Effect	
Zn1	2.07	His-54 NE2	His-54 ND1	Thr-53 OG1
Zn1	2.12	His-56 ND1	His-56 NE2	Wat’ O, Arg-163 O
Zn1	2.06	His-131 NE2	His-131 ND1	Arg-159 O
Zn1	2.62	Asp-150 OD2		
Zn1	1.94	Wat1 O		
Zn2	2.34	Asp-58 OD2		
Zn2	2.06	His-59 NE2	His-59 ND1	Asp-28 OD2
Zn2	2.05	Asp-150 OD2		
Zn2	2.03	His-191 NE2	His-191 O	Ser-189 OG
Zn2	2.00	Wat1 O		
Zn2	3.52	Wat2 O		

**Table 4:** Structurally aligned SLG binding residues in BLEG-1, YcbL, and GloB.

BLEG-1	YcbL	GloB
His-131 (Loop10) Hydrophobic interaction with Cys-moiety of SLG	His-132 (Loop8) Hydrophobic interaction with Gly-moiety of SLG	His-110 (Loop7) Hydrophobic interaction with Glu-moiety of SLG
Phe-153 (Loop12) Hydrophobic interaction with Gly- and Glu-moieties of SLG	Phe-154 (β10) Hydrophobic interaction with Gly-moiety of SLG	Phe-130 (β11) Hydrophobic interaction with Glu-moiety of SLG
Ile-157 (Loop12) Formation of hydrogen bond with –COOH in Gly-moiety of SLG	Val-158 (β11) Formation of hydrogen bond with –COOH in Gly-moiety of SLG	Cys-134 (β12) Formation of hydrogen bond with –COOH in Glu-moiety of SLG
Gly-158 (Loop12) Hydrophobic interaction with Gly-moiety of SLG	Gly-159 (Loop11) Hydrophobic interaction with Gly-moiety of SLG	Gly-135 (Loop9) Hydrophobic interaction with Glu-moiety of SLG
Arg-159 (Loop12) Formation of hydrogen bond with –CO in Gly- and Cys-moieties of SLG	Arg-160 (Loop11) Formation of hydrogen bond with –COOH in Gly-moiety of SLG	Arg-136 (Loop9) Formation of hydrogen bond with –COOH in Glu-moiety of SLG
His-191 (Loop14) Hydrophobic interaction with Glu-moiety of SLG	His-192 (Loop12) Formation of hydrogen bond with –COOH in Glu-moiety of SLG	His-165 (Loop11) Hydrophobic interaction with Gly-moiety of SLG

## Data Availability

Crystal structure of BLEG-1 presented in this study is openly available in the Protein Data Bank upon publication at https://doi.org/10.2210/pdb7EV5/pdb (accessed on 11 June 2021), PDB ID: 7EV5.

## Supplementary Materials

The following are available online at https://www.mdpi.com/article/10.3390/ijms22179377/s1.
