# Supplementary material for: Dual Activity BLEG-1 from Bacillus lehensis G1 Revealed Structural Resemblance to B3 Metallo-β-Lactamase and Glyoxalase II: An Insight into Its Enzyme Promiscuity and Evolutionary Divergence
Source: Int J Mol Sci. 2021 Aug 29;22(17):9377. doi: 10.3390/ijms22179377 (PMC8431146; doi:10.3390/ijms22179377)
Supplement: Supplementary file 1 [file ijms-22-09377-s001.zip › ijms-1322405-supplementary.pdf]

Supplementary materials

Table S1. Representative enzymes from each family of the metallo-hydrolase-like MBL-fold protein superfamily reported by Daiyasu et al (2001) [1], Bebrone (2007) [5], and Palzkill (2013) [11].

Member of Metallo-hydrolase-Like MBL-Fold Protein Superfamily	Protein Name	Organism	Accession	
			Genbank Accession Number	PDB ID
B1 MBL	BcII	*Bacillus cereus*	-	1BMC
	CcrA	*Bacteroides fragilis*	-	1ZNB
	IMP-1	*Pseudomonas aeruginosa*	-	1DD6
	BlaB	*Chryseobacterium meningoseptica*	-	1M2X
	SPM-1	*Pseudomonas aeruginosa*	-	2FHX
	NDM-1	*Klebsiella pneumonia*	-	3ZR9
	VIM-1	*Pseudomonas aeruginosa*	-	5N5G
	GIM-1	*Pseudomonas aeruginosa*	-	2YNT
	DIM-1	*Pseudomonas stutzeri*	-	4WD6
	TMB-1	*Achromobacter xylobacter*	-	5MMD
B2 MBL	CphA	*Aeromonas hydrophila*	-	1X8G
	Sfh-1	*Serratia fonticola*	-	3SD9
B3 MBL	L1	*Stenotrophomonas maltophilia*	-	1SML
	FEZ-1	*Legionella gormannii*	-	1K07
	BJP-1	*Bradyrhizobium japonicum*	-	3LVZ
	AIM-1	*Pseudomonas aeruginosa*	-	4AWY
	SMB-1	*Serratia marcescens*	-	3VPE
	GOB-18	*Elizabethkingia meningoseptica*	-	5K0W
Glyoxalase II (GLXII)	GloB	*Salmonella enterica subsp. enterica* serovar typhimurium	-	2QED
	YcbL	*Salmonella enterica*	-	2XF4
N-acyl-L-homoserine lactonase (AHL)	AiiA	*Bacillus thuringiensis*	-	2A7M
Flavodiiron proteins	ROO	*Desulfovibrio gigas*	-	1E5D
Cleavage and polyadenylation specificity factors	CPSF-73	*Homo sapiens*	-	2I7V
Arylsulfatase	SdsA1	*Pseudomonas aeruginosa*	-	2CG3
5′-exonuclease	Exo1	*Homo sapiens*	NP_666226.1	-
Ribonuclease	Tm	*Thermotoga maritima*	-	1WW1

Member of Metallo-hydrolase-Like MBL-Fold Protein Superfamily	Protein Name	Organism	Accession	
			Genbank Accession Number	PDB ID
Cyclic nucleotide phosphodiesterase	ZipD	*Escherichia coli*	-	2CBN
Insecticide hydrolase	MPH	*Pseudomonas sp* WBC-3	-	4LE6
Proteins required for natural transformation competence	ComA	*Neisseria gonorrhoeae*	P51973	-
Choline-binding protein	CbpE	*Streptococcus pneumoniae*	-	1WRA

Table S2. Comparison of enzyme kinetics data of several B3 MBLs and GLXIIs towards ampicillin and SLG respectively.

**B3 MBL**	**K_M (mM)**	**k_{cat} (s^{-1})**	**k_{cat}/K_M (mM^{-1} s^{-1})**
L1 [57]	3.0×10^{-1}	580	1.9×10^{3}
AIM-1 [58]	4.1×10^{-2}	594	1.4×10^{3}
FEZ-1 [59]	> 5	> 5.5	1.1×10
**GLXII**	**K_M (mM)**	**k_{cat} (s^{-1})**	**k_{cat}/K_M (mM^{-1} s^{-1})**
GloB [26]	2.95×10^{-1}	209.9	7.1×10^{2}
YcbL [35]	5.0×10^{-1}	0.9	1.8
A. thaliana GLXII [25]	3.91×10^{-1}	129	3.3×10^{2}

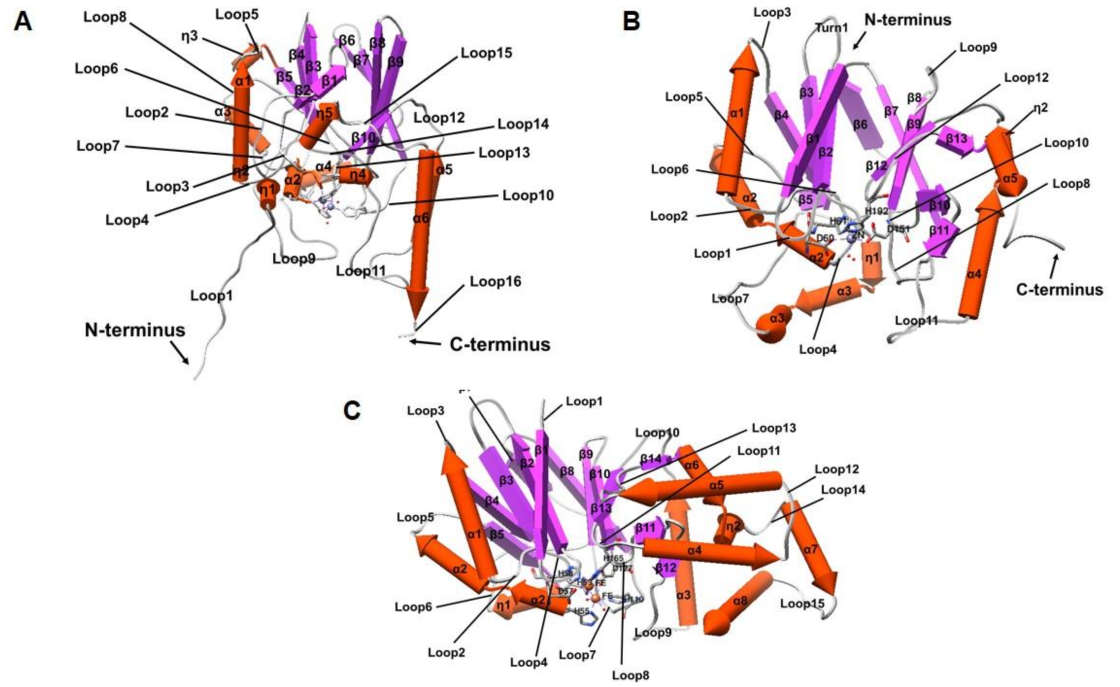

Figure S1. Crystal structure of (**A**) L1 MBL (PDB ID: 1SML, chain A), (**B**) YcbL (PDB ID: 2XF4, chain A) and (**C**) GloB (PDB ID: 2QED, chain A) with labelled secondary structure elements.
